# Application of an Improved Dual-Branch Model Based on Multi-Scale Feature Fusion in Fracture Surface Image Recognition

**DOI:** 10.3390/ma18225233

**Published:** 2025-11-19

**Authors:** Fei Gao, Denghui Wang, Fulai Yang, Mingping Zhou, Yuan Li, Zhen Zheng, Jianpeng Shi, Zheng Zhang

**Affiliations:** 1 School of Materials Science and Engineering, Beihang University, Beijing 100191, China; gaofei520@buaa.edu.cn (F.G.); zhoump@buaa.edu.cn (M.Z.); 18811327152@163.com (Y.L.); zhengzhen@buaa.edu.cn (Z.Z.); zy2401231@buaa.edu.cn (J.S.); 2 Taiyuan Heavy Industry Co., Ltd., Taiyuan 030027, China; wangdh@buaa.edu.cn

**Keywords:** SEM images, multi-scale feature fusion, interpretability analysis, improved dual-branch model, fracture surface image recognition

## Abstract

In order to improve the recognition accuracy and model interpretability of metal fracture scanning electron microscope (SEM) images, this research presents an improved dual-branch model (IDBM) based on multi-scale feature fusion. This model employs VGG19 and Inception V3 as parallel branches to separately extract local texture features and global semantic features. Furthermore, it integrates channel and spatial attention mechanisms to enhance the responsiveness of discriminative regions. By integrating dual-branch features using a fixed fusion ratio of 0.8:0.2, the model was trained and validated on an image dataset comprising 800 representative fracture surface images across four categories: cleavage, dimple, fatigue, and intergranular fracture. The results indicate that under small-sample data conditions, the IDBM achieves a Validation Accuracy (Val ACC) of 99.50%, a Recall rate of 99.51%, and an Area Under The Curve (AUC) value of 0.9998, significantly outperforming single models and other fusion strategies. Through integration with class activation mapping (CAM) and feature space visualization analysis, the model exhibits strong interpretability. Furthermore, scale adaptability tests reveal that IDBM maintains stable recognition performance across a magnification range of 100 to 10,000 times, and identifies the optimal observation magnification ranges for the four types of fractures.

## 1. Introduction

As the most intuitive and information-rich element in failure analysis, the accurate identification of fracture surface morphology plays a critical role [1]. It exerts a direct impact on the interpretation of failure mechanisms, root cause analysis, fracture mode classification, and the optimization of design and manufacturing processes [2,3]. Scanning electron microscope (SEM) images of metal fracture surfaces play an indispensable and critically important role in the process of failure analysis [4,5,6]. However, the identification of fracture surfaces traditionally depends on the expertise of experienced professionals. Despite variations in external conditions, chemical composition, and microstructure, the microscopic morphology of metal fractures can be classified into a few fundamental types, including cleavage, dimple, fatigue, and intergranular [7]. However, the microscopic morphologies in practical SEM images are not always typical [8,9]. When faced with a large volume of SEM images, the inefficiency and high degree of subjectivity associated with manual analysis have hindered the standardization and scalability of failure analysis processes. The core advantage of the machine-learning approach lies in its ability to learn from data and quantitatively assess characteristic and statistical features, thereby establishing a consistent set of criteria for judgment. The application of this method will simultaneously enhance efficiency and accuracy, ultimately achieving standardization in the fractographic recognition process [10]. Furthermore, the accurate determination of fracture types serves as the decisive basis for diagnosing the root cause of failure.

In recent years, image-based automatic recognition technology has exhibited significant application potential, driven by the rapid advancements in computer vision and deep learning techniques [11]. Convolutional neural networks (CNNs), which possess strong capabilities in feature extraction and pattern recognition, have been effectively utilized in research related to metal fracture surfaces [12,13]. Papia et al. [14] are revolutionizing SEM-based porous material analysis by automating pore size distribution measurement, pore shape classification, and image denoising, while also revealing future challenges and development directions for this field. Sun et al. [15], based on SEM/TEM image, found that a lightweight deep learning framework enables rapid and accurate (86.2%) nanoparticle segmentation and statistical analysis on embedded devices, demonstrating strong agreement with manual results. Nigam et al. [16] have successfully applied even simple CNN models to the automatic recognition of metallic microstructures with high accuracy. Mishra et al. [17] compared the accuracy of multiple CNN models but also utilized saliency maps to reveal the models’ decision-making mechanisms, providing insights for selecting reliable and interpretable algorithms under limited data conditions. Chen et al. [18] designed an encoder–decoder architecture, graph attention, and multi-scale fusion modules, which achieve high-accuracy segmentation with limited annotated samples, providing critical insights for revealing material structural transformation mechanisms. Wu et al. [19] improved the texture feature extraction of images, introduced a wireless transmission system, and replaced the empirical Ridgelet-2DPCA-based fracture recognition algorithm. These enhancements collectively improved the accuracy, efficiency, and flexibility of fracture detection. Wang et al. [20] proposed the N-Net model architecture for the classification and recognition of metal fracture images. They evaluated its performance by comparing the recognition accuracy with that of traditional machine learning algorithms on a metal fracture dataset, thereby validating the effectiveness of the N-Net-C model. Tsopanidis et al. [21] explored the potential of unsupervised machine learning in the field of fracture mechanics, specifically employing a convolutional CNN and the t-SNE dimensionality reduction algorithm to classify and cluster SEM images of fracture surfaces. This approach enabled the successful classification and clustering of SEM images of tungsten heavy alloys with varying tungsten contents. Yan et al. [22] significantly enhanced the accuracy of metal fracture type recognition by integrating features from multiple deep learning models, namely VGG16, VGG19, and ResNet50. However, the fracture surface morphology is highly complex and demonstrates pronounced multi-scale characteristics [23]. Particularly under complex operational conditions or mixed failure modes, distinguishing distinct features becomes increasingly challenging [24]. Furthermore, fracture images obtained in real industrial settings are often affected by challenges such as noise interference, scale variations, and partial occlusions. These complexities place significantly greater demands on the model’s robustness and generalization capacity.

Deep learning offers a novel methodology for automated fracture surface recognition. However, its application is constrained by several challenges. Existing deep learning models predominantly rely on single-scale convolutional kernels and related feature extraction or fusion techniques [25,26,27]. These approaches are thus limited in their ability to capture both fine textural details and high-level semantic features across fracture surfaces of varying complexity. Furthermore, the performance of current feature fusion methods significantly decreases when applied to small-scale datasets, which is considered in the context of deep learning for image recognition. And this in this study is the metal fracture SEM image dataset with 800 total images, with 200 images in each category. And the interpretability of model decisions remains poor, which hinders trust and adoption among materials scientists. To achieve this objective, this study proposes an improved dual-branch model (IDBM) that collaboratively extracts both texture and global morphological features through multi-scale branches. The model incorporates channel attention and spatial attention modules to emphasize discriminative regions, and employs a feature fusion strategy tailored for small-scale datasets. By integrating Class Activation Mapping (CAM) visualization with feature space evolution analysis, the model elucidates the underlying basis of its decision-making process [28].

## 2. Materials and Methods

### 2.1. Dataset

**Figure 1:**
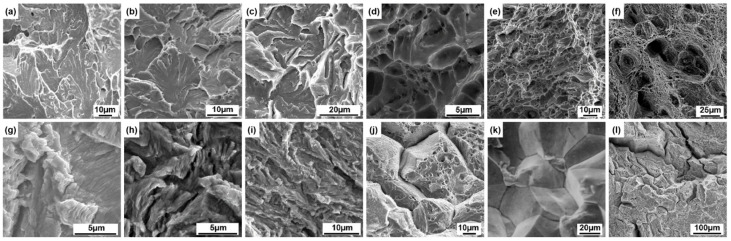
Datasets: (**a**–**c**) SEM images of cleavage fracture surfaces; (**d**–**f**) SEM images of dimple fracture surfaces; (**g**–**i**) SEM images of fatigue fracture surfaces; (**j**–**l**) SEM images representing intergranular fracture surfaces.

This paper has constructed four datasets as shown in Figure 1. These include fracture surface images of the hot-rolled Q345 steel and the as-cast and rolled states of ZL114 materials, as well as images obtained from publicly available books [23], doctoral and master’s theses [29,30], journals [31,32,33,34,35], and databases. The heat treatment parameters for the as-rolled ZL114 material are an entry temperature of 500 °C and an exit temperature of 230 °C. All material tests were conducted at room temperature. Among them, the tensile rate for the uniaxial tensile test was set at v = 1 mm/min. The fatigue test applied a preload of 0.5 KN, the stress waveform is a sine wave, the stress ratio is 0.1, and the frequency is 20 Hz. Pictures of the fracture surfaces were taken after the tests using a JSM-6010 scanning electron microscope (JEOL, Akishima, Japan) to obtain SEM images. The images are classified into four fracture types: cleavage, dimple, fatigue, and intergranular, with each category comprising 200 images, totaling 800 images. Among them, the images captured from the fracture surfaces of the tests account for approximately 70%. To evaluate the model’s image magnification performance, we used a dataset with the following images. These included 80,000-h service T92/Super304H steel welded joints and tensile fracture surfaces at room temperature and 650 °C. We also examined X65 steel fatigue fracture surfaces under sinusoidal wave conditions of −0.14 to −1.4 kN at 10 Hz. Additionally, we examined the fracture surfaces of X80 steel welds subjected to −10 °C impact.

### 2.2. Benchmark Model Selection

Considering the issue of sample size and following multiple comparisons, it was ultimately determined to adopt the VGG19 and Inception V3 models as the benchmark models for image feature extraction. These models are mature convolutional neural network (CNN) architectures with good performance in various image recognition tasks. They have complementary characteristics. VGG19 has a deep and uniform structure, excelling in local texture extraction [36]. Inception V3 has a multi-branch design, which enables it to capture features at multiple scales. These make them suitable candidates for constructing a dual-branch network to handle the complex, multi-scale nature of fracture surface images [37].

#### 2.2.1. VGG19

**Figure 2:**
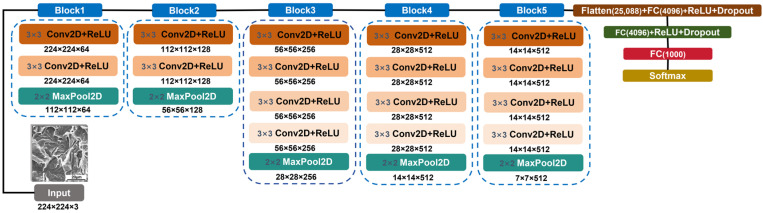
Architecture of the VGG19 Model. Note: The model adopts stacked 3 × 3 Conv2D kernels to expand the receptive field, with MaxPool2D layers for feature down-sampling and FC layers for final classification. Conv2D represents the two-dimensional convolution layer. MaxPool2D represents the two-dimensional maximum pooling layer. ReLU (Rectified Linear Unit) is an activation function. 3 × 3 represents that the size of the convolution kernel is 3 × 3. 224 × 224 × 64 represents the spatial dimensions of the feature map. The first two numbers represent the pixel values of the height and width of the output image, and the third number refers to the number of channels of the feature map. FC(4096) represents a fully connected layer that outputs 4096 neurons. Softmax represents a mathematical function.

The VGG19 architecture, proposed by Simonyan and Zisserman, is a landmark model in deep learning that demonstrates the importance of network depth. Its structure mainly consists of the stacking of 3 × 3 small convolutional kernels [25]. By replacing large convolutional filters with multiple successive small convolutions, it effectively reduces the number of parameters while preserving the receptive field and improving the capacity of the model for nonlinear representation [38]. The architecture is illustrated in Figure 2. The model, pre-trained on the ImageNet dataset, demonstrates strong feature extraction capabilities, making it particularly well-suited for analyzing image data with complex texture patterns and multi-scale morphological characteristics, such as SEM fracture surface images [39]. Its deep convolutional architecture enables the hierarchical extraction of discriminative features, ranging from low-level edges and textures to high-level semantic representations [4,40]. This allows the effective capture of both local and global patterns in characteristic microstructures, such as dimples, cleavage steps, and fatigue striations, on fracture surfaces. Furthermore, by leveraging transfer learning, VGG19 can effectively mitigate the overfitting issue arising from the limited availability of material image data, thereby enhancing the robustness and generalization capability of feature representations [41]. This makes it particularly well-suited for automatic recognition and classification tasks in metal fracture defect analysis.

#### 2.2.2. Inception V3

**Figure 3:**
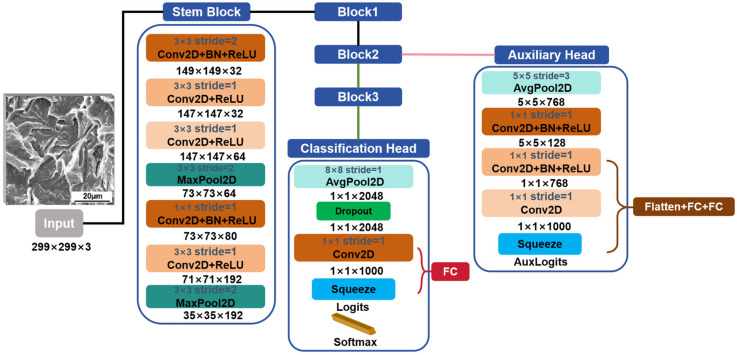
Architecture of the Inception V3 Model. Note: The Stem Block is the initial feature extraction module, while the auxiliary head and classification head form a dual-branch classification structure. Through multiple parallel paths, it extracts features of different scales. Conv2D represents the two-dimensional convolution layer. MaxPool2D represents the two-dimensional maximum pooling layer. AvgPool2D represents two-dimensional average pooling. ReLU (Rectified Linear Unit) is an activation function. 3 × 3 represents that the size of the convolution kernel is 3 × 3. 149 × 149 × 32 represents the spatial dimensions of the feature map. The first two numbers represent the pixel values of the height and width of the output image, and the third number refers to the number of channels of the feature map. FC represents a fully connected layer.

The core innovation of Inception V3 resides in its implementation of a multi-branch convolutional architecture. By utilizing multi-scale convolutional kernels (e.g., 1 × 1, 3 × 3, 5 × 5) and incorporating techniques like factorized convolutions and asymmetric convolutions, the model effectively captures multi-scale features, thereby maintaining a balance between representational power and computational efficiency [37]. This architectural design is depicted in Figure 3. In the analysis of SEM fracture images, the Inception V3 architecture effectively captures multi-level visual features ranging from microscopic local textures like fatigue striations and intergranular grain boundaries to macroscopic regional patterns such as dimple arrangements and intergranular sugar-like structures [42,43,44]. This makes it particularly well-suited for visual analysis tasks characterized by significant scale variations and structural complexity, while maintaining robust and discriminative feature representation even under limited data conditions [45].

### 2.3. IDBM

**Figure 4:**
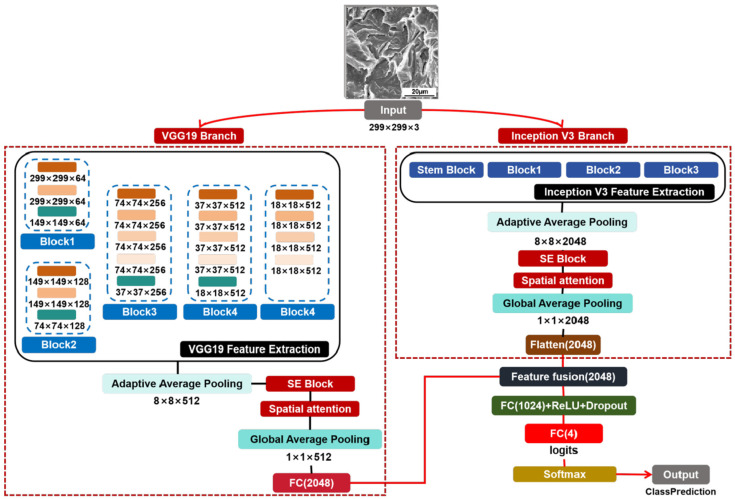
Architecture of IDBM. Note: The color coding of core modules (e.g., Conv2D, MaxPool2D, AvgPool2D) in this figure is consistent with that in Figure 2 (VGG19) and Figure 3 (Inception V3), where detailed explanations of the colored modules and their abbreviations are provided.

This study proposes a dual-branch network architecture based on VGG19 and Inception V3, with the overall structure illustrated in Figure 4. The core design principle of this architecture is to employ two pre-trained models with different structures and complementary strengths in feature extraction as parallel pathways for independent image feature extraction. Subsequently, an attention mechanism module is integrated into each pathway to amplify salient information. Finally, the enhanced features are fused to construct a recognition model with improved discriminative capability for SEM fracture morphology analysis.

Specifically, the input image is first uniformly resized to 299 × 299 pixels during preprocessing and then simultaneously fed into two fully parallel processing branches. Branch one employs the VGG19 architecture as the feature extraction backbone, with the original classification head removed and the final max pooling layer omitted to produce a high-dimensional feature map (F_vgg_). Branch two utilizes the Inception V3 model, with its classification head removed, as the primary feature extraction backbone, generating a high-dimensional feature map (F_inc_). Subsequently, following the output from the feature extractor in each branch, spatial adaptive average pooling is applied separately to F_vgg_ and F_inc_. The pooled features are then sequentially fed into the attention module. This module first employs the squeeze and excitation (SE) Block to learn the importance weights of feature channels, thereby recalibrating the channel-wise representations of F_vgg_ and F_inc_. Next, the spatial attention (SA) Block is utilized to learn the significance of different spatial positions, enabling recalibration of the spatial dimensions. Finally, feature maps enhanced with refined attention are obtained. Subsequently, global average pooling (GAP) is applied independently to the feature maps of both branches to generate one-dimensional feature vectors. To ensure dimensional consistency between the vectors, the output from the VGG19 branch is projected through a fully connected layer, after which feature fusion is performed. The fused comprehensive feature vector is fed into a classifier, which comprises a fully connected layer, a ReLU activation function, a dropout layer, and a final fully connected layer with an output dimension of 4. The classification result is then obtained through a softmax activation function, yielding the predicted category of the input image. Softmax is a mathematical function that can convert a set of arbitrary real numbers into a set of probability values, with the sum of these probability values equaling 1. It is particularly suitable for multi-classification problems and can intuitively provide the model’s confidence level for each class [46].

### 2.4. Experimental Setup

The experiments were conducted on a system equipped with a 12th Gen Intel^®^ Core™ i7-12700H processor, manufactured by ASUSTeK Computer Inc., Taipei, China (2.30 GHz), 16 GB of RAM, and an NVIDIA GeForce RTX 4060 Laptop GPU (8 GB VRAM), NVIDIA Control Panel version 8.1.969.0 (Santa Clara, CA, USA). The implementation was carried out in Python 3.10.9. For each dataset, 80% of the samples were randomly allocated to the training set, with the remaining 20% reserved for validation. This split was performed prior to all experimental procedures. To ensure the statistical significance and robustness of the report results, each experiment was repeated five times, and we calculated the average value and standard deviation to eliminate the randomness of a single result. And the data results have been recorded in the performance indicators of Table 1. In the final stage of evaluating the model’s adaptability to the scale of SEM images, an independent dataset was employed for testing.

## 3. Results

### 3.1. Key Requirements for Feature Extraction

SEM reveals distinct morphological characteristics of the four fundamental types of fracture surfaces, namely cleavage, fatigue, dimple, and intergranular, which enable their definitive identification (Figure 1a–l). The cleavage fracture surface exhibits dendritic patterns, formed by the convergence of cleavage steps across adjacent grains. Its core morphological attributes include river-like, tongue-like, fan-shaped, and fishbone patterns [47] (Figure 1a–c). The dimple fracture surface exhibiting dimples is characterized by the presence of clusters of honeycomb-like depressions (Figure 1d–f). Based on the stress conditions, these dimples can be categorized into three types: equiaxed dimples formed under uniform tensile stress, elongated parabolic-shaped shear dimples, and tear dimples developed under non-uniform deformation. The edges of the dimples appear bright, while the bottoms appear dark. Additionally, bright spots corresponding to second-phase particles are commonly observed at the centers of the dimples [48]. A characteristic feature of a fatigue fracture surface is the presence of parallel striations, where each individual striation corresponds to a single loading cycle. These are referred to as fatigue striations [49] (Figure 1g–i). The intergranular fracture surface exhibits a rock sugar-like stacked morphology along clearly defined grain boundaries (Figure 1j–l). The grain corners appear brighter, while the grain faces display contrast variations between light and dark regions, with these differences attributed to changes in crystallographic orientation [50].

#### 3.1.1. Texture Extraction Results

**Figure 5:**
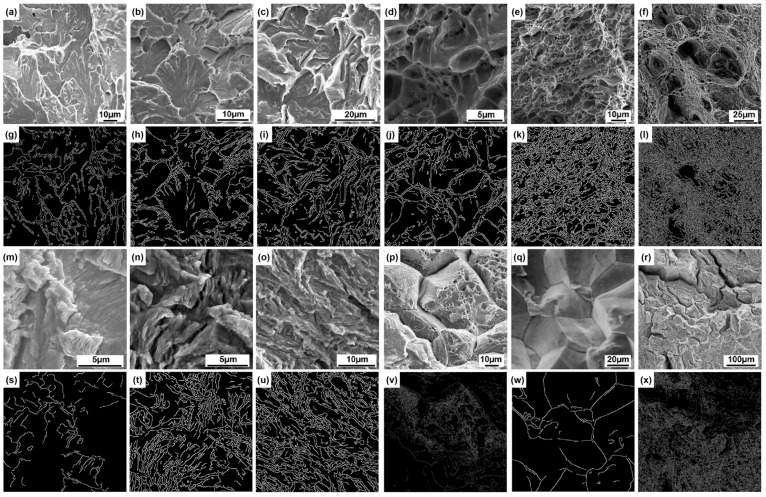
Texture Extraction Results: (**g**–**i**) correspond to the images (**a**–**c**); (**j**–**l**) correspond to the images (**d**–**f**); (**s**–**u**) correspond to the images (**m**–**o**); (**v**–**x**) correspond to the images (**p**–**r**), respectively.

Based on the four types of fracture surface morphology described above, texture feature extraction was performed on the fracture surfaces, as illustrated in Figure 5. The core logic of texture extraction primarily involves partitioning the image into blocks and analyzing the edge density within each block. Regions exhibiting edge density higher than the global average are classified as texture regions and assigned a white label, while areas with lower density are classified as non-texture regions and labeled as black. The extraction results indicate that the texture of cleavage fracture surfaces (Figure 5a–c,g–i) exhibits a high degree of consistency with the morphological characteristics of cleavage fracture surfaces. Cleavage fracture surfaces exhibit high-contrast linear edges in the image due to the presence of parallel striations formed by cleavage steps and steep edges resulting from microscopic height differences. In contrast, the texture extraction performance for the other three fracture types (dimple, fatigue, and intergranular) is constrained by several intrinsic limitations. These arise due to variations in dimple density, pronounced contrast fluctuations in fatigue striations, and the frequent coexistence of multiple fracture morphologies in intergranular surfaces. Such complexities, often resulting from material heterogeneity, complex loading histories, and environmental interactions, can lead to partial loss of characteristic texture information or the introduction of extraneous noise in the feature extraction process.

#### 3.1.2. Three-Dimensional Transformation Results

**Figure 6:**
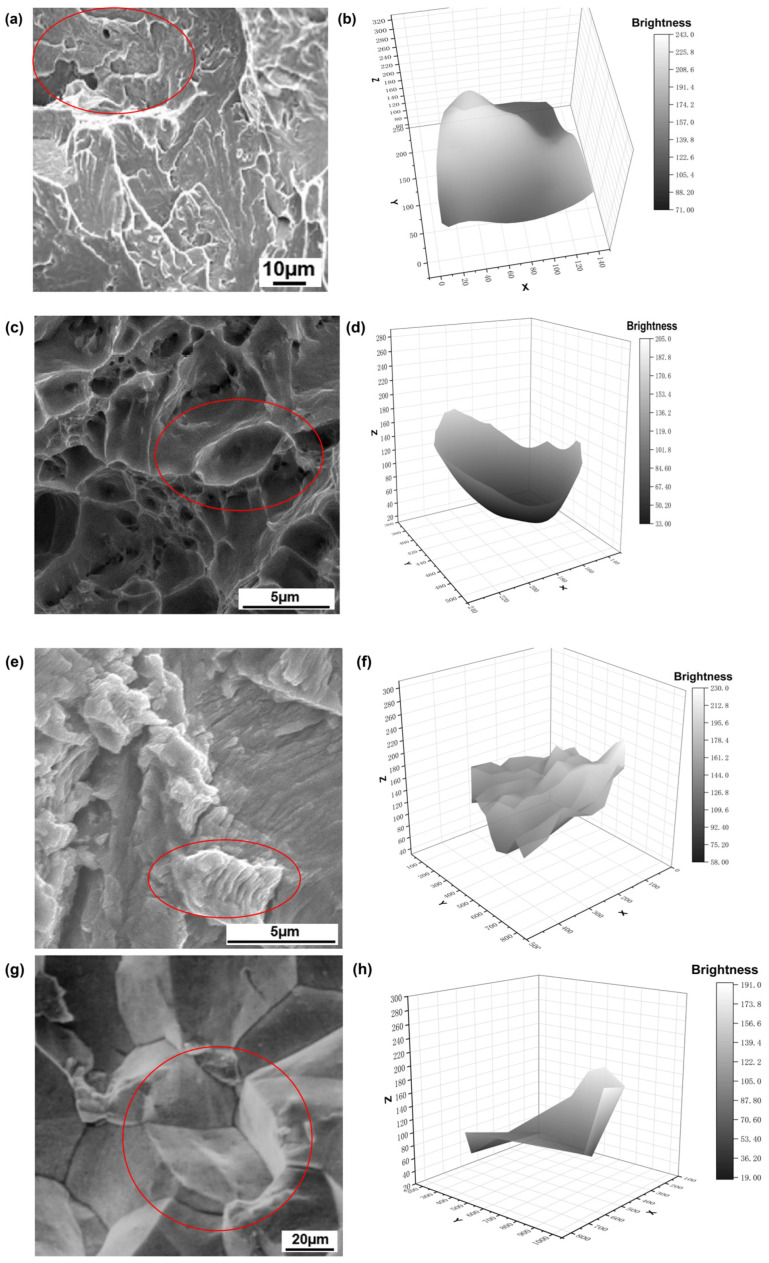
Three-Dimensional transformation results of the regions circled in red in (**a**), (**c**), (**e**), and (**g**), shown in (**b**), (**d**), (**f**), and (**h**), respectively.

Traditional SEM produces two-dimensional grayscale images that represent projections of three-dimensional fracture morphologies from a fixed observation angle. In this study, a novel approach based on three-dimensional grayscale image transformation was developed, in which grayscale projection techniques were employed to reconstruct the physical topography of fracture surfaces. The method for three-dimensional grayscale image transformation uses Python code to extract the pixel values of the selected area image, convert them to brightness values using the formula (Equation (1)), and finally plot the points in a three-dimensional coordinate system to obtain the result. This method significantly enhanced the interpretability and accuracy of identifying cleavage, dimple, fatigue, and intergranular fracture types. As shown in Figure 6, the X and Y coordinates correspond to the spatial location of pixel points within the image, while the Z-axis indicates the transformed brightness value of each pixel. The brightness values of the three-dimensional transformation results for all four fracture types were normalized to a common scale, enabling direct and quantitative comparison of feature height variations in three-dimensional space. This brightness value is derived by applying a specific conversion formula (Equation (1)) to the original pixel value at the corresponding pixel point. The original pixel value is obtained using a dedicated transformation algorithm, which is achieved through Python code, by extracting the RGB channel values of the selected area. The theoretical basis for this conversion lies in the photopic visual characteristics of the human eye, wherein three types of retinal cone cells exhibit distinct spectral sensitivities, as quantified by the CIE 1931 standard colorimetric system. According to this model, the human visual system is approximately twice as sensitive to green light as to red light and five times more sensitive to green than to blue [51]. Based on these empirical results, the International Commission on Illumination (CIE) formulated rigorous mathematical expressions for the computation of the luminance component, derived from extensive chromaticity matching experiments [52]. In engineering applications, the NTSC television standard offers a practical approximation of the CIE standard, providing an optimal trade-off between computational efficiency and accuracy in visual perception. Owing to their computational simplicity, the NTSC coefficients are extensively utilized in the field of image processing for luminance component calculations [53]. The formula for calculating the luminosity component (Z) is as follows:

(1) Z=0.299R+0.587G+0.114B

where R, G, and B are the pixel values of the red, green, and blue channels, respectively, each ranging from 0 to 255 [54].

The results demonstrate that the three-dimensional transformation of the cleavage fracture surface (Figure 6b) exhibits brightness values spanning from 93 to 243—a range of approximately 150 units. The surface is predominantly composed of bright regions, with the interiors of the river patterns corresponding largely to flat zones, consistent with the morphological attributes of cleavage steps. When interpreted alongside the texture extraction results derived from the corresponding SEM image, these findings indicate that the dominant morphological signature of this fracture type is characterized by textural line-like structures. Therefore, the proposed extraction principle addresses both linear textural structures, such as cleavage and step formations, as well as flat regions. To effectively capture these features, it is advised to combine small convolution kernels (e.g., 3 × 3), which excel at detecting local details, fine textures, edges, and corners, with larger kernels capable of integrating contextual information over broader spatial scales. Additionally, stacking multiple small kernels can approximate the receptive field of a larger kernel, thereby facilitating effective feature extraction from flat regions while maintaining computational efficiency.

In contrast to the transformed three-dimensional representation of the cleavage fracture surface, the transformation results of the other three fracture types exhibit more distinct and intuitively discernible morphological features. The dimple fracture surface image is characterized by the combination of multiple bowl-shaped structures, with an individual dimple exhibiting a morphology similar to that of a bowl-shaped structure (Figure 6d). The brightness values of these features span from 33 to 205, covering a range of approximately 172 units. The majority of the area occupies the lower brightness range, consistent with concave pit morphologies, while the elevated edges correspond to brighter values, indicative of dimple rims. The overall morphology of the dimple fracture surface is characterized by a high density of dimples arranged in an aggregated, honeycomb-like structure with sharply defined edges. Compared to the other three types of fracture surfaces, this structure exhibits the darkest values in the dimple bases and lacks virtually any flat or smooth regions. Therefore, our feature extraction principle for dimpled fracture surfaces is founded on a multi-scale framework. This entails identifying individual bowl-shaped units locally, analyzing their honeycomb-like arrangement structurally, and integrating depth-related morphological information globally. Small 3 × 3 convolutional kernels are primarily employed to capture the fine edge features of individual dimples, including intensity transitions and arcuate contours, given their small size of approximately 5 μm or less (Figure 6c). However, a single small kernel is often insufficient to encompass an entire dimple edge; thus, stacking two to three such layers is implemented to progressively integrate local responses into complete bowl-shaped structures. At the mesoscale, medium-sized kernels (5 × 5) are introduced to model the honeycomb-like topology formed by adjacent dimples, thereby capturing their spatial arrangement and collective morphology. At a deeper processing level, progressively expanding the receptive field to achieve full-field coverage is critical. Although stacking small convolutional kernels can theoretically enlarge the receptive field, practical coverage is often constrained by non-uniform weight distribution. Moreover, while deep hierarchical abstraction via successive small-kernel convolutions enhances feature learning, it also leads to the loss of fine-grained spatial details. This reduced sensitivity to precise pixel-level relationships hinders the accurate perception of mutual positioning and connectivity among dimples within honeycomb-like structures. Consequently, the effective extraction of dimple fracture features from SEM images necessitates the integration of larger convolutional kernels (e.g., 7 × 7) to compensate for these limitations and capture broader contextual cues.

The SEM image of the fatigue fracture surface (Figure 6f) reveals periodic alternation of contrasting light and dark bands, indicative of characteristic fatigue striations. The brightness values of these features span from 100 to 230, covering a range of approximately 130 units. Notably, the amplitude of brightness variation remains relatively low, with no discernible plateau regions observed. The fracture morphology is further marked by the presence of similarly aligned, parallel boundaries, consistent with the typical features of fatigue crack propagation. Therefore, in shallow network regions, it is advisable to use strip-shaped convolutional kernels to conform to parallel boundary structures. In deeper layers, however, larger convolutional kernels are recommended to directly capture cross-period luminance oscillations, thereby avoiding phase blurring that can result from cascading multiple small-kernel convolutions.

In the SEM image of the intergranular fracture surface, grain boundary positions display pronounced contrast variation, marked by well-defined edges alongside extended planar regions (Figure 6h). The morphology of individual grains exhibits a folded, paper-like structure, with brightness values ranging from 51 to 191—a range of approximately 140 units. The ratio of bright to dark regions is nearly 1:1, indicating a balanced distribution of steep-faced and shallow-angled features across the fracture surface. This suggests the absence of a predominant faceting orientation, with neither extensive flat areas nor concentrated clusters of steep cliffs observed. When multiple grains are interconnected, they collectively exhibit a characteristic pattern comprising zigzag lines interspersed with small flat regions. Therefore, it is recommended to employ non-symmetric kernels that align with the linear characteristics of grain boundaries, in order to prevent the inappropriate activation of isotropic kernels in flat regions. In deeper layers, the utilization of larger convolution kernels is advised to capture the contrast between adjacent light and dark grains, thereby improving the model’s discriminative capability.

### 3.2. Comparison of Feature Fusion Strategies

The multi-branch feature fusion strategies primarily encompass direct concatenation, weighted concatenation, adaptive concatenation, and Feature Pyramid Network (FPN). The direct concatenation is a feature fusion method of sequential connection. It connects feature vectors from different branches along the channel dimension without weight adjustment [55]. Weighted concatenation assigns fixed weights to the features of each branch and highlights the main information through linear fusion [56]. Adaptive concatenation is achieved by using a learnable network module to dynamically adjust the fusion weights based on the input image features, thereby enhancing the adaptability to complex data [57]. The Feature Pyramid Network performs fine-level fusion at the two-dimensional feature map level, and the purpose is to integrate low-level detailed features and high-level semantic features. Thereby, it will enhance the recognition ability for objects with scale variations [58]. To evaluate the performance of these feature fusion approaches, the following metrics are employed: training and validation loss values, training and Validation Accuracy, Recall rate, and AUC value. The formula for calculating the loss value is as follows:

(2) $$Loss=-\frac{1}{N}\times \sum \left[y_{i}\times \log p_{i}+\left(1-y_{i}\right)\times \log\left(1-p_{i}\right)\right]$$

where N is the total number of training samples, $y_{i}$ is the true label for the *i*-th sample, and $p_{i}$ is the corresponding predicted probability. The loss function directly quantifies the discrepancy between the predicted and actual values. The training loss indicates the degree of fit achieved during the training process; a lower training loss signifies a better fit to the training data. Validation loss quantifies the generalization error of the model on the validation data; a lower value indicates higher predictive accuracy on the validation set. The training loss and validation loss values are evaluated and compared based on their minimum values.

The accuracy formula is as follows:

(3) $$Accuracy=\frac{M}{N_{total}}$$

where M is the number of correct predictions, $N_{total}$ is the total sample size. Training accuracy is a metric that quantifies the classification performance of the model on the dataset used for training. Calculating the average training accuracy rate helps reflect the overall learning capability of the model while mitigating the impact of fluctuations that may occur during individual training epochs. The computation of the average training accuracy rate provides insight into the overall learning capability of the model, while reducing the influence of stochastic variations that can arise during specific iterations of the training process. The Validation Accuracy rate indicates the classification performance of the model on the validation set, serving as a metric to evaluate the overall capability of the model in correctly identifying samples. Selecting the maximum Validation Accuracy for assessment can effectively illustrate the optimal performance the model is capable of achieving.

The Recall formula is as follows:

(4) $$Recall=\frac{TP}{TP+FN}$$

where TP is the true positives, and FN is the false negatives. Recall is a measure of the ability of the model to correctly identify positive samples. A high Recall indicates a low rate of false negatives, meaning that the model is effective at capturing the majority of relevant cases. This metric is especially critical in evaluating the performance of recognition models used for classifying fracture surface images.

The AUC value formula is as follows:

(5) $$AUC=\frac{\sum_{i=1}^{M}\sum_{j=1}^{N}I\left(P_{i}>P_{j}\right)}{M\times N}$$

where M is the number of positive samples, N is the number of negative samples, $P_{i}$ denotes the predicted score for the i-th positive sample, $P_{j}$ denotes the predicted score for the *j*-th negative sample, I is the indicator function that equals 1 if the condition is satisfied $P_{i}>P_{j}$, and 0 otherwise. The AUC value comprehensively considers the performance under different thresholds, and the maximum value represents the optimal overall discrimination level. The comparison results are shown in Table 1.

The validation metrics (Val Loss, Val ACC, Recall, AUC) are used to evaluate the generalization performance of the model. The minimum value of Train Loss and the average value of Train ACC are used as auxiliary indicators to analyze the learning ability of each model. Among all the evaluated methods, the fixed-ratio concatenation approach (0.8 F_vgg_ + 0.2 F_inc_) achieved the highest performance across all validation metrics. Specifically, it attained a Val ACC of 0.9950, significantly surpassing the performance of basic methods. Additionally, the Val Loss was the lowest at 0.0323, indicating the strong generalization capability of the model. Recall was recorded at 0.9951, and the AUC reached 0.9998, and the SD in the majority of the indicators is around the lowest value. Therefore, these demonstrate the exceptional ability of the model to recognize positive samples and its overall classification robustness. Among the basic methods, the FPN demonstrates the best performance, achieving the highest Val ACC (0.9775) and Recall (0.9826), along with the lowest Val Loss (0.0983). Its AUC (0.9983) is on par with that of direct concatenation. In contrast, adaptive concatenation yields the worst results, with the highest Val Loss (0.1115), the lowest Recall (0.9679), and AUC (0.9979), suggesting that its fusion strategy may introduce noise or instability. Direct concatenation exhibits moderate performance. In the fixed ratio scheme, the test results of most ratios are superior to those of the basic method, indicating that controlling the feature ratio can effectively enhance model performance. Particularly when the VGG19 branch is assigned a relatively higher weight ratio, the feature extraction capability of the final dual-branch model is further improved. The performance of the model is influenced by the proportion of the VGG19 branch. The optimal performance is observed when the proportion falls within the range of 0.5 to 0.9, during which the Val ACC stabilizes between 0.9800 and 0.9950. However, when the proportion drops below 0.4, model performance deteriorates, as evidenced by an increase in Val Loss and a decline in Val ACC to between 0.9675 and 0.9700. The likely reason is that the VGG19 branch captures more robust features. If the corresponding weights are excessively low, it may result in an imbalance in information distribution. The overfitting tendencies of various feature fusion strategies are assessed by analyzing the contrast of the loss function, with the core formula expressed as:

Overfitting risk = Val Loss − Train Loss
(6)

By comparing the differences, it can be observed that the fixed ratio feature concatenation method with a ratio of 0.8:0.2 exhibits the lowest risk of overfitting, suggesting that this model is most appropriate for the task of classifying small data volume fracture SEM images.

### 3.3. Model Evaluation

#### 3.3.1. Evaluation Metrics

**Figure 7:**
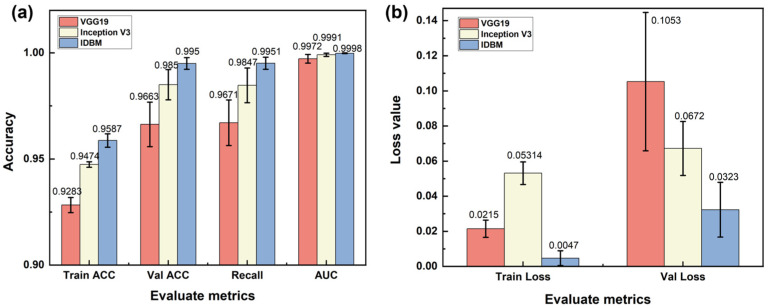
Bar charts of evaluation metrics: (**a**) Comparison of Train ACC, Val ACC, Recall, and AUC values among the three models; (**b**) Comparison of Train Loss and Val Loss of the three models.

To rigorously evaluate the effectiveness of the proposed multi-branch feature fusion model, a comprehensive performance assessment was carried out. A detailed comparative analysis was conducted between the IDBM and the baseline single-branch model. The evaluation criteria were based on the metrics outlined in Section 3.2, supplemented by the inclusion of confusion matrix diagrams for visual comparison. As shown in Figure 7a, on the training set, IDBM achieves superior performance, followed by Inception V3, while VGG19 exhibits the lowest accuracy. A similar trend is observed on the validation set, with IDBM maintaining the highest performance, again followed by Inception V3, and VGG19 continuing to yield the lowest results. This pattern suggests that increased model complexity correlates with enhanced performance on the validation set, and that IDBM possesses the strongest generalization capability among the three architectures. In terms of Recall, which measures the ability of the model to correctly identify positive instances, IDBM again surpasses the other models, demonstrating a superior capacity for detecting relevant samples. Furthermore, all three models achieve consistently high AUC, reflecting robust overall discriminative performance. As shown in Figure 7b, a lower Train Loss corresponds to a closer fit of the model to the training data. Among the evaluated models, IDBM achieves the smallest Train Loss, indicating the highest fidelity to the training set. Val Loss, which serves as a measure of generalization, is also minimized by IDBM. This result suggests that IDBM attains the lowest generalization error and exhibits the most stable performance on the validation set.

**Figure 8:**
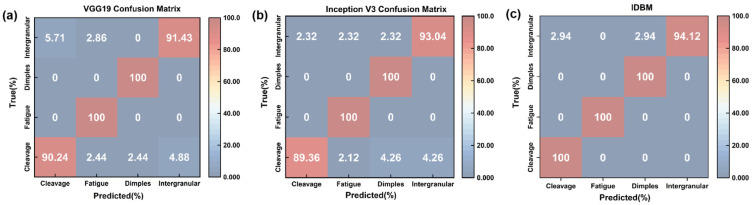
Confusion Matrix: (**a**) VGG19; (**b**) Inception V3; (**c**) IDBM.

As shown in Figure 8, the IDBM maintains consistently high classification accuracy across all categories, effectively reducing misclassification rates. VGG19 surpasses Inception V3 in identifying cleavage fracture surfaces, yet it underperforms slightly in recognizing intergranular fracture. Both reference models exhibit more pronounced misclassification tendencies compared to IDBM.

#### 3.3.2. Training Monitoring and Dimensionality Reduction Visualization

**Figure 9:**
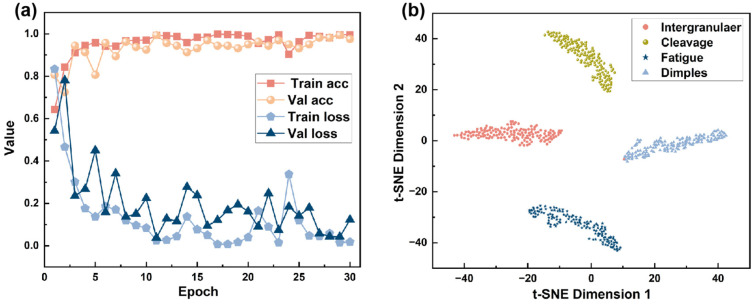
IDBM Evaluation Results: (**a**) Accuracy and loss curves for the training and validation sets; (**b**) t-SNE visualization.

As shown in Figure 9a, the training and validation performance of the IDBM indicates a steady improvement in Train ACC with increasing epochs. During the initial phase of approximately the first five epochs, accuracy increases rapidly, after which the rate of improvement declines and the metric gradually stabilizes, eventually converging to a value between 0.85 and 1. This behavior suggests that the model continuously enhances its performance on the training set until it reaches a satisfactory level of fit. Val ACC exhibits a general upward trajectory, though its rate of improvement remains more gradual than that of the Train ACC. After the 10th epoch, the Val ACC plateaus and continues to perform slightly below the Train ACC. This suggests that the performance of the model on the validation set gradually improves with the progression of training, and the overall fluctuation remains relatively small. The Train Loss decreases rapidly with an increase in the number of epochs. During the initial stages of training, the reduction in loss is particularly pronounced, after which the decline gradually tapers off and the loss eventually stabilizes at a value close to 0.05. This indicates that the model progressively learns the underlying patterns in the training data, resulting in an increasingly improved fitting performance and a continuous reduction in error. The Val Loss demonstrates an overall decreasing trend, although some fluctuations are observed during the training process. This variability may be attributed to the limited size of the validation dataset, which can lead to instability in the evaluation metrics.

The t-SNE is a dimensionality reduction technique capable of projecting high-dimensional data into two- or three-dimensional space. Its underlying principle involves preserving the local structure of the original high-dimensional data in the reduced-dimensional space, with the goal of maintaining the proximity of neighboring samples via probabilistic similarity matching. As a visualization diagnostic tool, t-SNE can be employed to assess whether a model has effectively learned meaningful category boundaries [59]. As shown in Figure 9b, the four fracture surface types form clearly segregated clusters, with only one instance from the intergranular fracture type being misclassified. This result indicates high separability among the four categories within the embedded feature space, with each forming well-separated clusters that demonstrate distinct boundaries. This finding indicates that the model effectively learns discriminative features across categories, thereby achieving robust performance in the classification task.

The explained variance ratio, also termed the variance contribution rate, indicates the proportion of total variance in the original dataset attributed to each principal component. It thus represents the amount of information preserved after dimensionality reduction. The proportion of variance explained by Principal Component 1 (PC1) reflects the percentage of total variance in the original dataset accounted for by this component, which corresponds to the dominant direction of variation. Similarly, the proportion explained by the Principal Component 2 (PC2) quantifies the variance captured along the second most influential direction of variability. The combined eigenvalues of the PC1 and PC2 correspond to the total variance preserved in the original data following projection into the two-dimensional subspace spanned by these components. This value serves as a crucial measure of how much information is preserved following dimensionality reduction. The results presented in Table 2 demonstrate that the IDBM achieves the highest cumulative explained variance ratio among the three models, indicating its superior ability to retain the majority of information within only two dimensions. Consequently, the information loss resulting from dimensionality reduction remains minimal, at approximately 12%. The features extracted by the IDBM exhibit a high degree of concentration, with the majority of information distributed along a dominant direction. As a result, the first two principal components are able to retain the vast majority (88.18%) of the information, demonstrating significantly higher efficiency in dimensionality reduction compared to VGG19 (60.79%) and Inception V3 (51.13%). VGG19 exhibits a strong dominant pattern, whereas the contributions of subsequent components are relatively minor. And the feature distribution of Inception V3 is more dispersed, making it less amenable to effective compression using only a small number of principal components.

### 3.4. Feature Extraction Visualization

CAM is designed to improve the interpretability of deep learning models, particularly in image recognition tasks. It generates a visual heatmap that delineates the regions of an input image most relevant to the prediction of the model for a given class. Areas with high activation intensity indicate strong contributions to the predicted outcome and are considered salient cues for the target category. Conversely, regions with low activation exert little to no influence on the classification decision [60].

**Figure 10:**
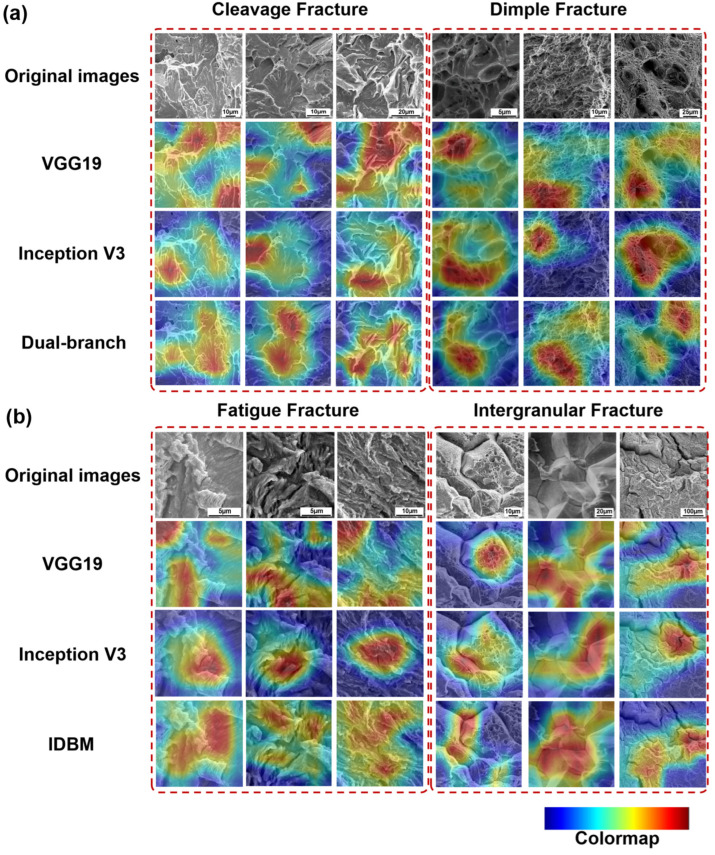
CAMs from three models for four sample types, obtained from SEM images: (**a**) The CAMs from cleavage fracture and dimple fracture; (**b**) The CAMs from fatigue fracture and intergranular fracture.

The CAM results for the four types of fracture surfaces across the three models are presented in Figure 10. In the CAMs, regions with a redder color indicate better image recognition performance, whereas regions with a bluer color indicate poorer recognition performance. The results indicate that the VGG19 model exhibits superior performance in extracting textural features. Specifically, it demonstrates accurate localization of texture line regions in cleavage SEM images and covers extensive areas of fatigue striation patterns in selected fatigue fracture samples. For intergranular SEM images, the model captures grain boundary features with relatively comprehensive coverage. However, in cases involving more complex intergranular structures, occasional misidentification of grain boundary characteristics may occur. The Inception V3 model exhibits enhanced capability in capturing high-level semantic features and processing relatively complex intergranular structures. However, it demonstrates limited effectiveness in both regional coverage and feature localization accuracy when applied to cleavage SEM images with distinct textural patterns. By comparison, the IDBM improves both the coverage and accuracy of feature extraction across all four fracture surface types. It enables precise identification of river patterns in cleavage structures, bowl- and honeycomb-shaped morphologies in dimples, and accurate characterization of fatigue striation patterns. Furthermore, it exhibits enhanced capability in localizing grain boundary regions—both in isolated intergranular SEM images and within more complex intergranular fracture surfaces. In conclusion, the CAM result graph demonstrates that IDBM can relatively accurately capture the features of the four types of fracture SEM images, thereby providing a more comprehensive explanation for the superior recognition performance of the model across these image categories. This result is of great significance. Users can verify through the CAM whether the recognition positions of the model are based on the accurate features in various fracture types. Moreover, in scenarios involving complex or mixed fracture patterns, these CAMs serve as diagnostic auxiliary tools, indicating the key areas that affect the classification results, thereby guiding users to conduct subsequent manual inspections. Finally, the CAM can also highlight the characteristics of different fracture types, which is helpful for the training of analysts.

### 3.5. Model Adaptability to SEM Image Scale

The IDBM employed in this study integrates the detailed feature extraction capability of VGG19 with the multi-scale abstraction ability of Inception V3, thereby theoretically achieving superior and stable discriminative performance in handling scale variations, which enhances recognition robustness. To verify this feature, this study performed an evaluation based on the constructed multi-material cross-scale test dataset, which includes the fracture surface images of T92/Super304H steel welded joints, X65 steel, and X80 steel welds.

The variability in scale (magnification) during SEM imaging significantly challenges the robustness and generalization capability of the model. The visual characteristics of the same target, including texture details, contrast relative to the background, field of view, and structural integrity, exhibit notable variations across different magnification levels. While a model may demonstrate strong performance during training at a specific scale, its effectiveness can significantly decline when applied to a markedly different scale, for instance, from low-magnification overall analysis to high-magnification detail examination. This scale sensitivity restricts the applicability of the model across diverse and dynamic research environments. Consequently, a systematic evaluation and analysis of deep learning adaptability of the model to varying scales in scanning SEM images are essential to enhance their practical utility and establish robust, automated SEM image analysis workflows.

**Figure 11:**
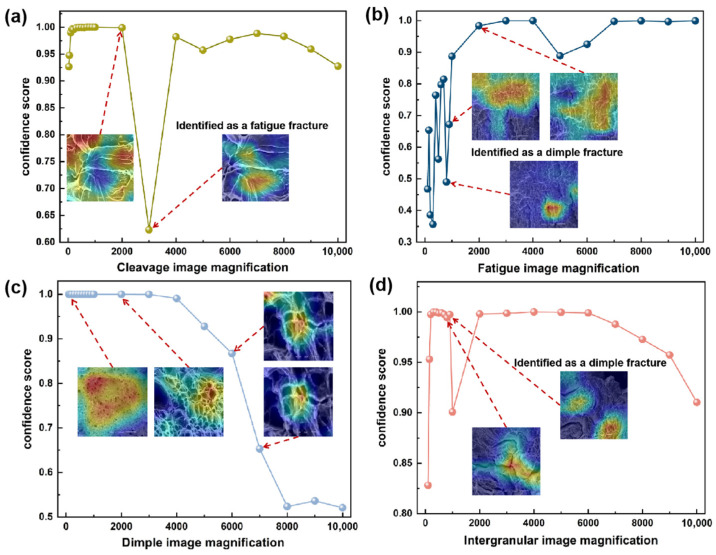
Recognition performance of the IDBM on fracture surface SEM images at different magnification levels: (**a**) cleavage, (**b**) fatigue, (**c**) dimple, (**d**) intergranular identification results, respectively.

As shown in Figure 11, the optimum magnification levels were evaluated for the four fracture surface types. Specimens excluded from the training and validation sets were subjected to fractographic analysis using SEM. Images were captured across a magnification range from 100× to 10,000× and subsequently fed into the model for evaluation. In the cleavage fracture scale adaptability test, the dataset of X80 fracture surface images was selected as the test material. The results indicate that the cleavage fracture surface demonstrates the highest recognition accuracy within a magnification range of 100× to 2000× (Figure 11a). When the magnification exceeds 2000×, the absence of a complete river pattern structure in the image may lead the model to misclassify the surface as a fatigue fracture. This suggests that the accurate identification of cleavage fracture surfaces is highly dependent on the full visibility of characteristic morphological features. It is therefore recommended to perform recognition at magnifications below 2000×, although the optimal magnification may also vary depending on the material’s actual grain size and the imaged area.

In the scale adaptability assessment for fatigue fracture, the test material comprised a dataset of X65 steel fatigue fracture images. Fatigue fracture features were reliably identified within a magnification range of 900× to 10,000× (Figure 11b). At magnifications above 900×, the IDBM achieved fracture type identification with a confidence level of approximately 0.5. When magnification exceeded 2000×, the confidence level increased to over 0.89. These results suggest that this fracture type is more easily identifiable at higher magnifications, where fatigue striations become increasingly distinct. However, excessive magnification may lead to erroneous classification of a cleavage fracture as a fatigue fracture, as illustrated by the 3000× example in Figure 11a. Thus, the recommended optimal magnification range for reliable identification lies between 900× and 3000×.

For the scale adaptability evaluation of dimple fracture surfaces, a dataset of T92/Super304H steel welded joints, high-temperature tensile fracture specimens was employed. Dimple features can be reliably identified from SEM images within a magnification range of 100× to 10,000× (Figure 11c). However, when magnification exceeds 7000×, the recognition confidence falls below 0.7. Excessive magnification adversely affects the discernibility of the honeycomb-like dimple morphology, thereby reducing the stability of feature recognition. Thus, for dimple fracture surfaces, the recommended optimal magnification range lies between 100× and 7000× to ensure reliable identification.

For the scale adaptability assessment of grain boundary fractures, a dataset comprising T92/Super304H steel welded joints, steel room-temperature tensile fracture specimens was utilized. Recognition performance demonstrated relatively effective results within a magnification range of 100× to 900× (Figure 11d). This identification method relies primarily on the global morphology of grain boundaries. At magnifications exceeding 900×, local microstructural features become disproportionately emphasized, potentially leading to misinterpretation. It is therefore recommended that identification be conducted at low to medium magnification levels, with an optimal operational range between 100× and 900× for accurate classification.

Overall, the IDBM demonstrates consistent discriminative performance across varying magnification levels, confirming the effectiveness and generalization capacity of its multi-scale feature integration strategy. It should be noted that the above conclusions are drawn from the fracture surfaces of the specific materials investigated in this study. The efficacy of feature recognition is highly dependent on microstructural integrity, image noise, and grain size. Excessive magnification narrows the field of view, resulting in an incomplete representation of characteristic features, while overly low magnification introduces information redundancy and compromised detail clarity. In practical implementations, magnification should be adjusted adaptively according to the grain size of the material and the quality of the acquired images. The ranges identified in this study may provide a practical reference for such applications.

## 4. Discussion

### 4.1. Analysis of Performance Advantages and Multi-Scale Feature Fusion Mechanism

The VGG19 branch employs stacked small convolutional kernels to attain a wide receptive field, enabling effective extraction of river-like, tongue-like, and fishbone-like textures in cleavage fracture SEM images. Moreover, it achieves higher accuracy in identifying bright edge textures surrounding individual dimples within ductile fracture surfaces. The stacking of small convolutional kernels enables the capture of overall texture orientation within both localized areas and slightly larger spatial regions. Medium-sized and large convolutional kernels are employed to extract continuous dimple morphology and honeycomb topological features from the SEM images of fatigue fracture surfaces, as well as the bright and dark contrast characteristics of adjacent grains in the SEM images of intergranular fracture surfaces. The parallel texture of fatigue striations is extracted using a rectangular kernel, which is capable of matching long-range continuous feature stripes, enhancing the edge response in the target orientation, and suppressing noise in the perpendicular direction. By employing asymmetric kernels to extract textures of intergranular boundaries with varying orientations, the model leverages the spatial asymmetry of these kernels, which are specifically designed to capture asymmetric image features. The confusion matrix results indicate that Inception V3 outperforms VGG19 in the classification of intergranular boundaries, whereas it demonstrates relatively lower accuracy in classifying cleavage types. The core advantage of IDBM lies in its multi-scale feature fusion mechanism and three-dimensional shape-guided network architecture. By effectively integrating the local texture sensitivity of VGG19 and the global topological perception capability of Inception V3, the model achieves superior generalization performance while substantially mitigating the risk of overfitting. Furthermore, the Inception V3 branch compensates for the VGG19 branch’s limitation in capturing inflection points along polylines. Based on the physical insights derived from the three-dimensional analysis of SEM images (such as the correlation between the depth of dimples and the Z-value gradient, as well as the correspondence between cleavage steps and flat bright regions), the convolutional kernel structure was specifically designed: small kernels (3 × 3) were primarily responsible for extracting cleavage textures and dimple edges; asymmetric kernels were employed to enhance the detection of intergranular boundaries; elongated kernels improved the identification of fatigue striations; and large kernels (7 × 7) were utilized to capture the honeycomb-like morphology of dimples and align with the periodic characteristics of fatigue striations. This morphological and network collaborative optimization mechanism has effectively addressed the limitations of traditional models in identifying complex fracture features.

IDBM demonstrates superior performance across key indicators compared to the single-branch benchmark model. The cumulative explained variance ratio of the principal components is markedly higher than that of VGG19 and Inception V3, indicating a more compact feature representation. Furthermore, the t-SNE visualization demonstrates that the feature distributions of the four fracture surface types are distinctly clustered. CAM further substantiates its discriminative superiority: IDBM accurately identifies critical regions such as cleavage river patterns and bright spots along the edges of dimples, whereas VGG19 tends to perform poorly in complex grain boundary environments. Although Inception V3 exhibits noticeable noise during texture extraction, the influence of its rectangular and asymmetric convolutional kernels enables it to achieve more accurate feature extraction for intergranular and certain fatigue fractures compared to VGG19. Based on the network architecture models of VGG19 and Inception V3, as well as the CAM results presented in Figure 10, it can be observed that the structural characteristics and feature extraction capabilities of these two models are largely complementary. Therefore, selecting them as benchmark models within the IDBM framework is well-justified. Furthermore, IDBM achieves optimal performance through the implementation of a fixed-ratio feature fusion strategy, specifically 0.8 VGG19 and 0.2 Inception V3. This particular ratio effectively balances the complementary nature of low-level texture features and high-level semantic topological features, while simultaneously minimizing noise interference and information redundancy. This proportionate combination adheres to the discrimination principle of fracture morphology, characterized by edge dominance and topological support. Under conditions of limited sample availability, it effectively mitigates the overfitting tendency of complex models at a fixed ratio while maximizing inter-class separability.

### 4.2. Value of Interpretable Mechanisms and Scale Adaptation for Analyzing Material Failure

The extraction of texture features and three-dimensional transformations constitutes the foundational basis for constructing a deep learning model aimed at identifying the types of SEM images of fracture surfaces, thereby directly informing the selection of the base model. In the method of three-dimensional transformations, the grayscale information from SEM images is converted into quantifiable topographic height features, as illustrated in Figure 6. This approach elucidates the direct correlation between fracture surface morphology and height features: dark regions corresponding to dimples represent low Z-value valleys, while flat areas associated with cleavage steps exhibit low brightness gradients. This methodology establishes a solid materials science foundation for the design of convolution kernels—such as bowl-shaped edge detection and grain boundary sharpening—thereby overcoming the limitations inherent in traditional “black box” models.

Furthermore, the scale adaptability tests (Figure 11) validated the robustness of the model over a magnification range of 100× to 10,000×, underscoring its practical utility. These results establish an optimal reference scale range for characterizing the four fracture surface types, which provides a theoretical basis for standardizing fractographic analysis and also furnishes empirical support for developing automated SEM inspection protocols. By dynamically adapting to grain size variations, such as through the application of higher magnifications for fine-grained materials, this approach effectively mitigates the problem of incomplete feature capture inherent in conventional fixed-magnification methods.

This study establishes a robust physical foundation and structural framework for developing a deep learning model to recognize fracture surface SEM images, employing two key techniques: texture feature extraction and three-dimensional morphological reconstruction. This approach substantially improves the interpretability of the model and practical applicability in engineering contexts. The edge density-based texture extraction method (Figure 5) effectively accentuates river patterns in cleavage fracture surfaces. However, it also reveals limitations in characterizing dimple structures, fatigue striations, and intergranular morphologies, which particularly include the model’s conventional susceptibility to noise interference and potential loss of localized structural detail. This discovery directly led to the incorporation of multi-scale and attention mechanisms into the dual-branch model, aiming to effectively integrate structural details with contextual semantics, thereby compensating for the limitations associated with relying solely on texture-based representations.

The three-dimensional topographic reconstruction based on the NTSC luminance conversion formula (Formula (1); Figure 6) enables the transformation of grayscale information in SEM images into height features with explicit physical interpretability, thereby establishing a quantitative mapping between pixel intensity and surface topography. For example, the extensive flat regions observed in cleavage fracture surfaces correspond to a relatively gradual variation in Z-value, whereas the pronounced undulations along the edges of dimples are represented by marked changes in brightness. Additionally, fatigue striations exhibit periodically alternating high and low undulation patterns. This morphology–feature mapping relationship not only establishes a materials science foundation for the design of convolution kernels—for instance, employing small convolution kernels to detect cleavage steps, utilizing asymmetric kernels to identify grain boundaries, and applying rectangular kernels to align with fatigue striations—but also fundamentally transcends the limitations of traditional deep learning “black box” models, thereby enabling the model’s decision-making process to be supported by traceable physical interpretations.

Based on this, the study further conducted a systematic scale adaptability test (Figure 11) to assess the model’s recognition performance and stability across varying magnification levels, ranging from 100× to 10,000×. The results demonstrate that IDBM maintains robust performance across varying magnification levels. However, its recognition capability remains strongly dependent on the morphological integrity of the fracture type. Specifically, cleavage and intergranular fractures require fully preserved macroscopic morphology and are thus better identified at low to medium magnifications (≤2000×). In contrast, fatigue and dimple fractures, which are characterized by distinctive local microfeatures, maintain high recognition confidence even at higher magnifications (e.g., 2000×–10,000×). This finding holds significant practical utility. Primarily, it can provide a theoretical basis for standardizing SEM image acquisition protocols tailored to different fracture types by recommending appropriate magnification ranges, thereby facilitating more consistent inspection procedures. Furthermore, the model can dynamically adapt its feature extraction strategy based on the actual image scale, thereby reducing issues such as feature omission or misinterpretation due to inappropriate magnification, which is particularly beneficial in multi-material contexts with substantial grain size variations.

Therefore, texture extraction and three-dimensional topography reconstruction not only provide a theoretical foundation for model structure optimization but also significantly enhance its interpretability and physical plausibility. Concurrently, the demonstrated scale adaptability of the system underscores the practical utility of the model and generalization capacity in actual industrial settings. The integration of these aspects allows IDBM to serve not only as a high-accuracy image classification tool but also as a semantically intelligent diagnostic system capable of interpreting materials science information. This dual functionality enables seamless incorporation into existing failure analysis workflows and provides a robust foundation for automated, high-throughput, and interpretable fracture surface analysis. In the future, a software interface can also be developed based on this for the analysis of fracture images, or combined with AI to create an answerable mode for fracture images. This will further enhance the efficiency and intelligence of fracture analysis, significantly lower the barrier for using our model, and enable high-throughput and standardized fracture analysis.

## 5. Conclusions

Focusing on fracture surface image data from small datasets comprising cleavage, fatigue, dimple, and intergranular fracture types, an IDBM is proposed. Through comprehensive evaluation of model performance, interpretability analysis of its decision-making mechanism, and scale adaptability testing across varying SEM image magnifications, the following conclusions are drawn:

1. This study presents an IDBM built upon the VGG19 and Inception V3 architectures. The model incorporates channel and spatial attention mechanisms and utilizes a multi-scale feature fusion strategy with a fixed ratio of 0.8:0.2. Its core structure consists of dual-branch parallel feature extraction, adaptive attention weighting, and a fusion mechanism based on global average pooling. The architecture ultimately performs high-accuracy classification of four fracture types through a fully connected classification layer.
2. The IDBM employs a fixed-ratio multi-scale fusion strategy, which exhibits superior performance in fracture surface image recognition compared to conventional approaches such as direct concatenation, adaptive fusion, and pyramid feature fusion. The model achieves a Val ACC of 99.50%, a Recall of 99.51%, and an AUC of 0.9998. This strategy effectively reduces overfitting risks while enhancing the generalization capacity of the model and discriminative stability.
3. Through three-dimensional reconstruction of fracture surface SEM images, a quantitative mapping between pixel values and surface topography was established. The integration of this method with texture feature extraction significantly enhances the interpretability of the model. Furthermore, it provides a theoretical and empirical basis in materials science for designing convolutional kernels and selecting baseline models in novel algorithm development, thereby facilitating structural optimization and feature visualization in fracture surface recognition models.
4. Based on the scale adaptability tests, the optimal magnification ranges for the four fracture surface types are summarized as follows: cleavage fracture (≤2000×), fatigue fracture (900–3000×), dimple fracture (100–7000×), and intergranular fracture (100–900×). The experimental results demonstrate that the IDBM exhibits strong robustness across varying magnification levels. Furthermore, it remains essential to select a suitable imaging scale depending on the specific fracture type and the preservation state of its microstructural characteristics.

## Figures and Tables

**Table 1:** Comparison of Different Concatenation Methods and Weighting Strategies in Fixed-Ratio Feature Fusion.

Methods	Fixed Proportion		Train Loss ± SD	Train ACC ± SD	Val Loss ± SD	Val ACC ± SD	Recall ± SD	AUC ± SD	Overfitting Risk ± SD
Direct Concatenation	-	-	0.0150 ± 0.0081	0.9580 ± 0.0118	0.1000 ± 0.0300	0.9700 ± 0.0103	0.9741 ± 0.0091	0.9984 ± 0.0011	0.1121 ± 0.0599
Adaptive Concatenation	-	-	0.0100 ± 0.0070	0.9506 ± 0.0027	0.1115 ± 0.0300	0.9676 ± 0.0053	0.9679 ± 0.0046	0.9979 ± 0.0008	0.1106 ± 0.0417
FPN	-	-	0.0180 ± 0.0076	0.9154 ± 0.0218	0.0983 ± 0.0541	0.9775 ± 0.0105	0.9826 ± 0.0124	0.9983 ± 0.0013	0.0691 ± 0.0390
Fixed-Ratio Concatenation	0.9 F_vgg_	0.1 F_inc_	0.0040 ± 0.0049	0.9476 ± 0.0058	0.0627 ± 0.0190	0.9850 ± 0.0035	0.9853 ± 0.0037	0.9993 ± 0.0005	0.0832 ± 0.0283
	0.8 F_vgg_	0.2 F_inc_	0.0047 ± 0.0042	0.9587 ± 0.0031	0.0323 ± 0.0156	0.9950 ± 0.0028	0.9951 ± 0.0028	0.9998 ± 0.0002	0.0364 ± 0.0259
	0.7 F_vgg_	0.3 F_inc_	0.0054 ± 0.0025	0.9588 ± 0.0020	0.0560 ± 0.0275	0.9812 ± 0.0076	0.9823 ± 0.0071	0.9994 ± 0.0006	0.0778 ± 0.0450
	0.6 F_vgg_	0.4 F_inc_	0.0080 ± 0.0053	0.9600 ± 0.0024	0.0729 ± 0.0510	0.9838 ± 0.0095	0.9835 ± 0.0097	0.9989 ± 0.0014	0.0646 ± 0.0392
	0.5 F_vgg_	0.5 F_inc_	0.0056 ± 0.0036	0.9577 ± 0.0043	0.0799 ± 0.1580	0.9800 ± 0.0052	0.9807 ± 0.0054	0.9989 ± 0.0005	0.0674 ± 0.0206
	0.4 F_vgg_	0.6 F_inc_	0.0102 ± 0.0051	0.9573 ± 0.0032	0.1056 ± 0.0378	0.9700 ± 0.0081	0.9705 ± 0.0085	0.9976 ± 0.0014	0.1088 ± 0.0338
	0.3 F_vgg_	0.7 F_inc_	0.0090 ± 0.0084	0.9608 ± 0.0045	0.1019 ± 0.0285	0.9700 ± 0.0081	0.9695 ± 0.0103	0.9982 ± 0.0010	0.1031 ± 0.0323
	0.2 F_vgg_	0.8 F_inc_	0.0116 ± 0.0051	0.9598 ± 0.0030	0.1055 ± 0.0230	0.9700 ± 0.0068	0.9700 ± 0.0059	0.9980 ± 0.0009	0.1199 ± 0.0456
	0.1 F_vgg_	0.9 F_inc_	0.0064 ± 0.0058	0.9609 ± 0.0019	0.0976 ± 0.0306	0.9675 ± 0.0051	0.9681 ± 0.0045	0.9983 ± 0.0014	0.1230 ± 0.0579

Note: The evaluation metrics are defined as follows: Val Loss (Validation Loss) quantifies the generalization error on the validation set; Val ACC (Validation Accuracy) indicates the classification accuracy on the validation set; Recall measures the model’s ability to correctly identify samples; AUC (Area Under the Curve) represents the overall classification robustness across different thresholds. SD (Standard Deviation) measures the dispersion of data around the mean.

**Table 2:** Explained Variance Distribution by Model.

Models	PC1 Explained Variance Proportion (%)	PC2 Explained Variance Proportion (%)	PC1 and PC2 Explained Variance Proportion (%)
VGG19	56.97	3.82	60.79
Inception V3	30.03	21.10	51.13
IDBM	76.59	11.58	88.18

## Data Availability

The original contributions presented in this study are included in the article. Further inquiries can be directed to the corresponding author.
